# Oxytocin ameliorates impaired social behavior in a mouse model of 3q29 deletion syndrome

**DOI:** 10.1186/s13041-022-00915-w

**Published:** 2022-03-28

**Authors:** Tomoya Takemoto, Masayuki Baba, Kazumasa Yokoyama, Kohei Kitagawa, Kazuki Nagayasu, Yukio Ago, Kaoru Seiriki, Atsuko Hayata-Takano, Atsushi Kasai, Daisuke Mori, Norio Ozaki, Kazuhiro Takuma, Ryota Hashimoto, Hitoshi Hashimoto, Takanobu Nakazawa

**Affiliations:** 1 Laboratory of Molecular Neuropharmacology, Graduate School of Pharmaceutical Sciences, Osaka University, Suita, Osaka 565-0871 Japan; 2 Discovery Accelerator, Astellas Pharma Inc., Tsukuba-shi, Ibaraki 305-8585 Japan; 3 Department of Molecular Pharmacology, Graduate School of Pharmaceutical Sciences, Kyoto University, Sakyo-ku, Kyoto, 606-8501 Japan; 4 Department of Cellular and Molecular Pharmacology, Graduate School of Biomedical and Health Sciences, Hiroshima University, Minami-ku, Hiroshima, 734-8553 Japan; 5 Interdisciplinary Program for Biomedical Sciences, Institute for Transdisciplinary Graduate Degree Programs, Osaka University, Suita, Osaka 565-0871 Japan; 6 United Graduate School of Child Development, Molecular Research Center for Children’s Mental Development, Osaka University, Kanazawa University, Hamamatsu University School of Medicine, Chiba University and University of Fukui, Suita, Osaka 565-0871 Japan; 7 Department of Psychiatry, Nagoya University Graduate School of Medicine, Nagoya, Aichi 466-8550 Japan; 8 Brain and Mind Research Center, Nagoya University, Nagoya, Aichi 466-8550 Japan; 9 Department of Pharmacology, Graduate School of Dentistry, Osaka University, Suita, Osaka 565-0871 Japan; 10 Department of Pathology of Mental Diseases, National Institute of Mental Health, National Center of Neurology and Psychiatry, Kodaira, Tokyo, 187-8553 Japan; 11 Division of Bioscience, Institute for Datability Science, Osaka University, Suita, Osaka 565-0871 Japan; 12 Transdimensional Life Imaging Division, Institute for Open and Transdisciplinary Research Initiatives, Osaka University, Suita, Osaka 565-0871 Japan; 13 Department of Molecular Pharmaceutical Science, Graduate School of Medicine, Osaka University, Suita, Osaka 565-0871 Japan; 14 Laboratory of Molecular Biology, Department of Bioscience, Graduate School of Life Sciences, Tokyo University of Agriculture, 1-1-1 Sakuragaoka, Setagaya-ku, Tokyo, 156-8502 Japan

**Keywords:** 3q29 microdeletion, Autism spectrum disorder, Oxytocin, Paraventricular nucleus, Social behavior

## Abstract

Autism spectrum disorder (ASD) is a neurodevelopmental condition characterized by specific social symptoms, restricted interests, stereotyped repetitive behaviors, and delayed language development. The 3q29 microdeletion (3q29del), a recurrent copy number variant, confers a high risk for ASD and schizophrenia, and serves as an important pathological model for investigating the molecular pathogenesis of a large number of neurodevelopmental and psychiatric conditions. Recently, mouse models carrying a deletion of the chromosomal region corresponding to the human 3q29 region (Df/+ mice) were generated and demonstrated neurodevelopmental and psychiatric conditions associated behavioral abnormalities, pointing to the relevance of Df/+ mice as a model for these conditions with high construct and face validity. Currently, the molecular pathogenesis of these behavioral phenotypes in Df/+ mice remains unclear. The oxytocin (OXT) system plays a central role in social behavior across species and has a potential role in ASD. In this study, to elucidate the molecular mechanisms behind impaired social behavior in Df/+ mice, we investigated the possible involvement of OXT signaling in impaired social behavior in Df/+ mice. We demonstrated that OXT administration restored the impaired social behavior in Df/+ mice. We also demonstrated that the number of OXT-positive cells in the paraventricular nucleus (PVN) was significantly lower in Df/+ mice than in wild-type (WT) littermates. Consistent with this, the level of OXT peptide in the cerebral cortex of Df/+ mice was lower than in WT littermates. Our study may provide important insights into the molecular pathophysiological basis of neurodevelopmental and psychiatric conditions, including ASD.

**Supplementary information:**

The online version contains supplementary material available at 10.1186/s13041-022-00915-w.

## Introduction

Autism spectrum disorder (ASD) is a neurodevelopmental condition characterized by specific social symptoms, restricted interests, stereotyped repetitive behaviors, and delayed language development [1]; its incidence is increasing worldwide [2]. Although recent genetic studies have identified high-confidence ASD-associated genetic variants [3], the molecular pathogenesis of ASD remains unclear [1].

Recent genetic studies have demonstrated that recurrent copy number variants (CNVs) are associated with a high risk for ASD [4]. The 3q29 microdeletion (3q29del) is typically an approximately 1.6 Mb deletion and contains 21 protein-coding genes [5]. Although the incidence of 3q29del is low (1 in 30,000–40,000 birth), 3q29del confers high risk for ASD both in males (odds ratio, 24.6) and females (odds ratio, 41.8), as well as for schizophrenia [6]. These exceptionally high odds ratios suggest that the cellular and molecular etiology of patients with 3q29del is an invaluable clue for elucidating the complex mechanisms of ASD. Recently, mouse models carrying a heterozygous deletion of the chromosomal region corresponding to the human 3q29 region (deficiency (Df)/+ mice) were generated and showed various neurodevelopmental and psychiatric conditions-related behavioral abnormalities, including ASD-related behavioral abnormalities [7, 8]. This highlights the relevance of Df/+ mice as an ASD model as well as various neurodevelopmental and psychiatric conditions with significant construct and face validity. Currently, the molecular pathogenesis of ASD-related phenotypes in Df/+ mice remains unclear.

Recent studies have suggested that genetic variants of the oxytocin (OXT) receptor are associated with social behavior in humans and that impaired OXT signaling is linked to ASD [9]. In mice, targeted disruption of the *Oxt* or OXT receptor (*Oxtr*) gene impairs social behavior, suggesting that the OXT system plays an important role in social behavior across species [9]. Thus, focusing on the OXT system may provide clues to shed light on the molecular mechanisms regulating social behavior in mice. Currently, limited studies have shown a link between the OXT system and behavioral phenotypes in mouse models of ASD [for example see 10, 11]. In this study, to elucidate the molecular mechanisms behind impaired social behavior in Df/+ mice, we investigated the possible involvement of OXT signaling in impaired social behavior in Df/+ mice.

## Results

**Fig. 1:**
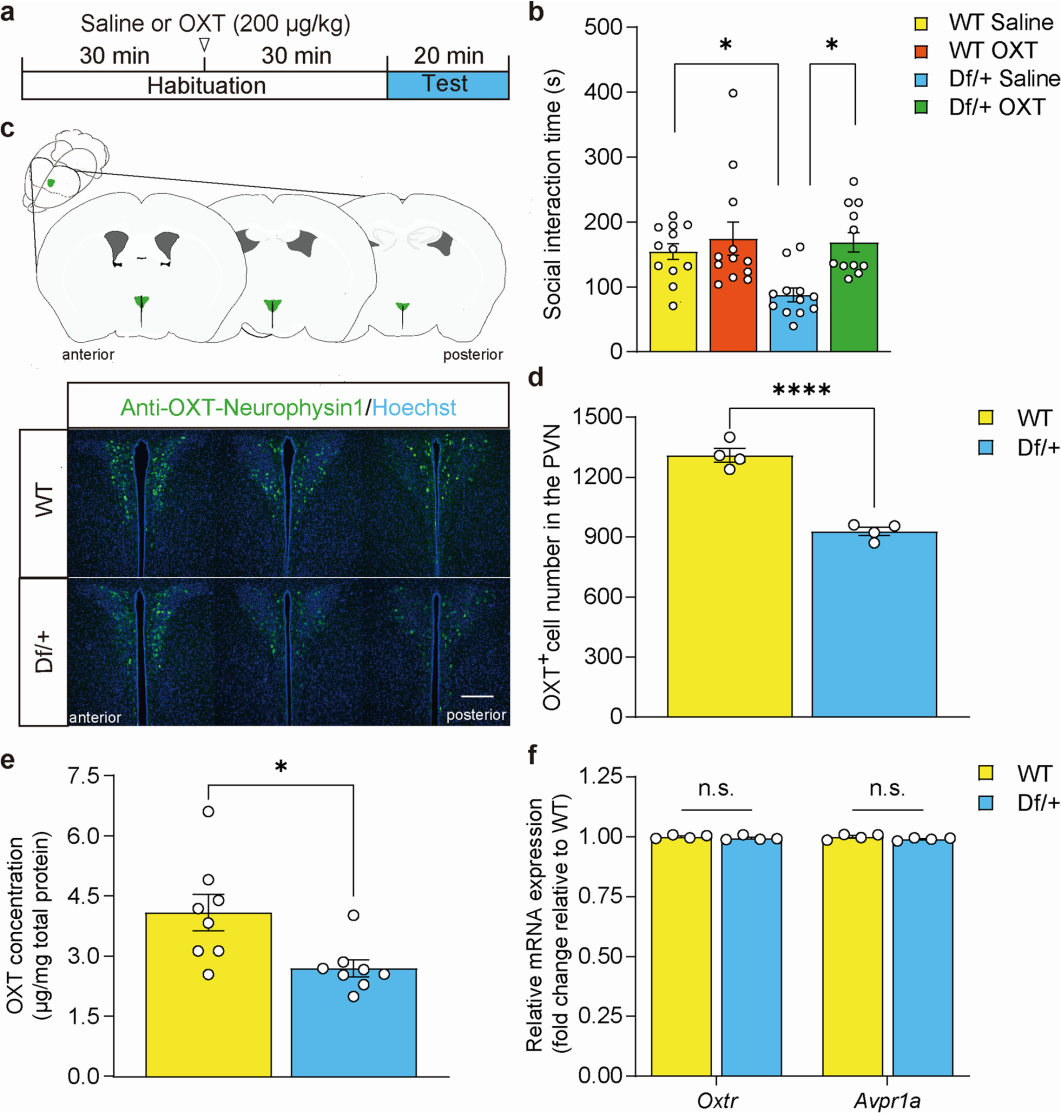
Administration of OXT improved impaired social interaction in Df/+ mice. **a** Timeline for the reciprocal social interaction test with OXT treatment. The test was carried out 30 min after administration of OXT (200 µg/kg) or saline. OXT, Oxytocin. **b** Social interaction time in the reciprocal social interaction test (each n = 12). The time spent sniffing, following, allo-grooming, and push-crawling of test mice toward the intruder was measured as social interaction time (two-way ANOVA for genotype, F_1,44_ = 8.84, P = 0.0048; for treatment, F_1,44_ = 4.61, P = 0.037; genotype × treatment interaction, F_1,44_ = 3.23, P = 0.079). WT, wild-type. **c** (*upper*) Schematic illustration of the PVN (green) and (*lower*) representative images of OXT-Neurophysin1 immunostaining (green) and hoechst33258 staining (blue) in the PVN of adult Df/+ mice and WT littermates. Scale bar, 100 μm. **d** Quantification of the number of OXT-positive cells in the PVN (each n = 4 mice). **e** ELISA quantification of the OXT concentration in the cerebral cortex extract (each n = 8 mice). **f** Quantitative real-time RT-PCR analysis of the expression levels of *Oxtr* and *Avpr1a* mRNA (each n = 4 mice). *Oxtr*, OXT receptor; *Avpr1a*, vasopressin receptor 1 A. Data are represented as the mean ± SEM. Statistical significance was analyzed by a two-way ANOVA, followed by Bonferroni Dunn *post hoc* tests (**b**) and Student’s *t*-test (**d**–**f**). *P < 0.05, ****P < 0.0001, n,s, not significant

To investigate whether oxytocin administration ameliorates impaired social behavior in Df/+ mice, we carried out a reciprocal social interaction test with the administration of 200 µg/kg of OXT (Fig. 1a). As we and other groups previously reported [7, 8], Df/+ mice treated with saline showed decreased social interaction time compared to that of WT littermates (Fig. 1b). We revealed that intraperitoneal administration of OXT improved impaired social interaction in Df/+ mice to a level similar to that in WT littermates (Fig. 1b). However, it did not have significant effect on the social interaction time in WT littermates at the concentration used (Fig. 1b).

To elucidate the molecular mechanism underlying the effect of OXT in Df/+ mice, we examined the abnormalities of OXT-related signaling pathways in Df/+ mice. We assessed the number of OXT neurons in the paraventricular nucleus (PVN), where OXT neurons are densely located [9], and found that the number of OXT-positive cells was significantly lower in Df/+ mice than in WT littermates (Fig. 1c and d). We assumed that the decreased number of OXT-positive cells in Df/+ mice may result in decreased OXT concentration in the cerebral cortex, where OXT signaling plays an important role in the regulation of social behavior. To probe this possibility, we quantified levels of OXT peptide in the cerebral cortex and found decreased levels of OXT peptide in the cerebral cortex of Df/+ mice as compared to that in WT littermates (Fig. 1e). Finally, we measured the expression of the *Oxtr* and vasopressin receptor 1 A (*Avpr1a*) mRNAs, which encodes for another receptor for OXT, in the cerebral cortex by quantitative real-time RT-PCR. Results showed that the expression levels of *Oxtr* and *Avpr1a* mRNAs were not significantly changed in Df/+ mice as compared to those in WT littermates (Fig. 1f).

## Discussion

Df/+ mice show social dysfunction, one of the major ASD-related behavioral phenotypes. However, the molecular pathophysiological mechanisms underlying these phenotypes remain largely unclear. In this study, we provide evidence suggesting that impaired OXT system is associated with social dysfunction in Df/+ mice. Specifically, our current results offer a new avenue for investigating the molecular mechanisms underlying the effect of 3q29del on social symptoms. Considering that the 3q29del confers an extremely high risk for ASD as well as various neurodevelopmental and psychiatric conditions [6], our study may provide important insights into the molecular pathophysiological basis of ASD as well as various neurodevelopmental and psychiatric conditions.

The molecular links between the OXT system and the 21 protein-coding genes in the 3q29 deleted region remain unknown. Among the 21 gene products, PAK2, which regulates actin cytoskeletal dynamics and dendritic spine morphology, is suggested to be mainly associated with ASD [10]. However, *PAK2* mRNA expression is decreased by OXT treatment in the hippocampus [11], suggesting that PAK2 might not be involved in the effect of OXT in Df/+ mice. In addition to *PAK2*, a recent study suggests that four genes, including *CEP19*, *SENP5*, *UBXN7*, and *WDR53*, out of the 21 protein-coding genes in the 3q29-deleted region may be primary ASD-associated genes [12]. Unraveling the precise molecular links between impaired OXT system and dysfunction of these possible ASD-associated gene products will help elucidate the molecular pathophysiological mechanisms of social dysfunction in Df/+ mice.

Several ASD mouse models, such as POGZ-Q1038R, Nlgn3^−/−^, and Cntnap2^−/−^ mice, have an impaired OXT system [13–15]. This suggests that, despite the etiological heterogeneity of ASD, an impaired OXT system may underpin the social symptoms in a subset of patients. Considering that the social symptoms in the patients with 3q29del may be distinct from those in idiopathic ASD patients [6] and that the social symptoms are common features of various neurodevelopmental and psychiatric conditions, further studies on the possible molecular links between an impaired OXT system and social symptoms may help advance a molecular mechanism-based stratification of groups of patient with various neurodevelopmental and psychiatric conditions as well as the development of molecular pathogenesis-based therapeutic interventions, extending well beyond OXT treatment.

## Supplementary Information

**Additional file 1.** Detailed materials and methods.

## Data Availability

All data and materials are available from the corresponding author upon reasonable request.

## Abbreviations

ASD: Autism spectrum disorder
CNV: Copy number variant
OXT: Oxytocin
PVN: Paraventricular nucleus
WT: Wild-type
