# Correction to: Oxytocin ameliorates impaired social behavior in a mouse model of 3q29 deletion syndrome

**DOI:** 10.1186/s13041-022-00920-z

**Published:** 2022-04-11

**Authors:** Tomoya Takemoto, Masayuki Baba, Kazumasa Yokoyama, Kohei Kitagawa, Kazuki Nagayasu, Yukio Ago, Kaoru Seiriki, Atsuko Hayata-Takano, Atsushi Kasai, Daisuke Mori, Norio Ozaki, Kazuhiro Takuma, Ryota Hashimoto, Hitoshi Hashimoto, Takanobu Nakazawa

**Affiliations:** 1 Laboratory of Molecular Neuropharmacology, Graduate School of Pharmaceutical Sciences, Osaka University, Suita, Osaka 565-0871 Japan; 2 Discovery Accelerator, Astellas Pharma Inc., Tsukuba-shi, Ibaraki 305-8585 Japan; 3 Department of Molecular Pharmacology, Graduate School of Pharmaceutical Sciences, Kyoto University, Sakyo-ku, Kyoto, 606-8501 Japan; 4 Department of Cellular and Molecular Pharmacology, Graduate School of Biomedical and Health Sciences, Hiroshima University, Minami-ku, Hiroshima, 734-8553 Japan; 5 Interdisciplinary Program for Biomedical Sciences, Institute for Transdisciplinary Graduate Degree Programs, Osaka University, Suita, Osaka 565-0871 Japan; 6 United Graduate School of Child Development, Molecular Research Center for Children’s Mental Development, Osaka University, Kanazawa University, Hamamatsu University School of Medicine, Chiba University and University of Fukui, Suita, Osaka 565-0871 Japan; 7 Department of Psychiatry, Nagoya University Graduate School of Medicine, Nagoya, Aichi 466-8550 Japan; 8 Brain and Mind Research Center, Nagoya University, Nagoya, Aichi 466-8550 Japan; 9 Department of Pharmacology, Graduate School of Dentistry, Osaka University, Suita, Osaka 565-0871 Japan; 10 Department of Pathology of Mental Diseases, National Institute of Mental Health, National Center of Neurology and Psychiatry, Kodaira, Tokyo, 187-8553 Japan; 11 Division of Bioscience, Institute for Datability Science, Osaka University, Suita, Osaka 565-0871 Japan; 12 Transdimensional Life Imaging Division, Institute for Open and Transdisciplinary Research Initiatives, Osaka University, Suita, Osaka 565-0871 Japan; 13 Department of Molecular Pharmaceutical Science, Graduate School of Medicine, Osaka University, Suita, Osaka 565-0871 Japan; 14 Laboratory of Molecular Biology, Department of Bioscience, Graduate School of Life Sciences, Tokyo University of Agriculture, 1-1-1 Sakuragaoka, Setagaya-ku, Tokyo, 156-8502 Japan

## Correction to: Molecular Brain (2022) 15:26 https://doi.org/10.1186/s13041-022-00915-w.

Following publication of the original article [1], a typesetting error was identified in the article title.

The article title was incorrectly published as: Molecular brain (micro report) oxytocin ameliorates impaired social behavior in a mouse model of 3q29 deletion syndrome.

The article title should read: Oxytocin ameliorates impaired social behavior in a mouse model of 3q29 deletion syndrome.

The error has been corrected and the original article has been updated. The publisher apologises to the authors and readers for the inconvenience caused by this mistake.
