# Supplementary material for: Oxytocin ameliorates impaired social behavior in a mouse model of 3q29 deletion syndrome
Source: Mol Brain. 2022 Mar 28;15:26. doi: 10.1186/s13041-022-00915-w (PMC8962454; doi:10.1186/s13041-022-00915-w)
Supplement: Supplementary file 1 — Additional file 1. Detailed materials and methods. [file 13041_2022_915_MOESM1_ESM.pdf]

Additional File 1

Detailed Materials and Methods

Animal experiments were performed in accordance with the guidelines for animal use issued by the Committee of Animal Experiments at Osaka University (#28-1-15).

Drug administration

Oxytocin (OXT) (Peptide Institute Inc., Osaka, Japan) was dissolved in saline (Otsuka Pharmaceutical Co., Ltd., Tokushima, Japan) at 20 μg/mL. OXT was intraperitoneally administered to mice at a volume of 10 mL/kg 30 min before the reciprocal social interaction test. The dose of OXT used in this study was determined based on previous studies (Neurmann et al., Psychoneuroendocrinol., 38:1985-1993 (2013); Galbusera et al., Neuropsychopharmacol., 42:1420-1434 (2017); Hara et al., Horm. behav., 96:130-136 (2017); Smith et al., Pharmacol. Res., 146:104324 (2019); Yamasue et al., Mol. Psychiatry, 25:1849-1858 (2020)).

Reciprocal social interaction test

Mice were housed in group cages with 4 animals per cage kept in environmentally controlled room with a 12-h light and dark cycle. For the social interaction test, 16-week-old male Df/+ mice and their WT littermates (C57BL/6J) were used in this study. The reciprocal social interaction test was carried out using a wooden box (22.5 × 33.8 × 14 cm) under white light (300 lux). Prior to the test, each test mouse was habituated to the test arena for 1h. OXT or saline was administered 30 minutes at the end of habituation. Then, a 7-week-old grouped-housed male mouse (C57BL/6J, Japan SLC, Shizuoka, Japan), socially naïve to the test mice, was used as an intruder mouse and placed in the test arena. The total amount of time that the test mouse spent sniffing, following,

allo-grooming, and push-crawling the intruder was manually measured over the entire experimental period (20 min). Behavioral analyses were performed in a completely blinded manner.

Immunohistochemistry

For immunohistochemistry, 12-week-old mice were deeply anesthetized and perfused with saline, followed by 4% paraformaldehyde (PFA) dissolved in phosphate-buffered saline (PBS). The brains were excised, post-fixed in 4% PFA at 4℃ overnight, and sectioned at a thickness of 30 μm using a cryostat (Leica, Wetzlar, Germany, CM1860). The brain slices were permeabilized with a blocking solution containing 5% bovine serum albumin and 0.3% Triton X-100 in PBS for 1 h at room temperature. Slices were then incubated with a blocking solution containing rabbit anti-oxytocin/neurophysin 1 (Abcam, #ab2078, 1:1000) at 4 ℃ overnight. The next day, slices were incubated with a blocking solution containing Alexa Fluor 488-conjugated goat anti-rabbit IgG (Life Technologies, CA, USA, #A-11008, 1:1000) and Hoechst 33258 dye (Calbiochem, CA, USA) for 1 h at room temperature. Images of the stained slices were obtained using a BZ-9000 microscope (Keyence, Osaka, Japan) and analyzed using ImageJ (NIH, MD, USA).

Enzyme-linked immunosorbent assay (ELISA) quantification of OXT

Brain dissection was performed between 10:00 and 12:00 hours. To measure the OXT level, slices of the cerebral cortex from 12–16-week-old mice were prepared using Brain Matrix (Brainscience-Idea, Osaka, Japan) and were preserved at -80 ℃ until measurement. On the day of measurement, tissues were homogenized with ×50 volume of RIPA buffer containing 1:100 protease inhibitor (Nacalai Tesque, Kyoto, Japan). Subsequently, lysates were centrifuged at 10,000 ×g for 15 min at 4 ℃, and the resultant supernatants were used for measurement with an

oxytocin ELISA kit (Enzo Life Sciences, Farmingdale, NY, USA). The absorbance was measured at 415 nm using an iMark microplate reader (Bio-Rad Laboratories, Inc. Hercules, CA, USA). Total protein concentration was measured using a BCA assay (Thermo Fisher Scientific, Waltham, MA, USA) per the manufacturer's instructions.

Real-time reverse transcription (RT)-PCR

Total RNA from the cerebral cortex of 12-weeks-old mice was prepared using the RNeasy Plus Mini Kit (QIAGEN, Hilden, Germany) according to the manufacturer's instructions. Total RNA was reverse-transcribed using SuperScript III (Life Technologies, CA, USA). Real-time RT-PCR was performed with TB Green Premix Ex Taq II (Takara Bio Inc., Shiga, Japan) using a CFX96 real-time PCR detection system (Bio-Rad Laboratories, CA, USA). The primers for *Oxtr*, *Avpr1a*, and *Gapdh* were as follows; *Oxtr* (forward primer sequence: 5′-GTGCAGATGTGGAGCGTCT-3′; reverse primer sequence: 5′-GTTGAGGCTGGCCAAGAG-3′), *Avpr1a* (forward primer sequence: 5′-CAATTTCGTTTGGACCGATT-3′; reverse primer sequence: 5′-TGTTCAAGGAAGCCAGTAACG-3′), and *Gapdh* (forward primer sequence: 5′-GTGTTCCTACCCCCAATGTG-3′; reverse primer sequence: 5′-TACCAGGAAATGAGCTTGAC-3′). The expression levels of *Oxtr* and *Avpr1a* were normalized to those of *Gapdh* and were determined using the $2^{-\Delta Ct}$ method.

Statistical analysis

The behavioral data were statistically analyzed using a two-way ANOVA, followed by Bonferroni–Dunn *post hoc* tests. The quantified data from immunohistochemistry, quantitative real-time RT-PCR, and ELISA were statistically analyzed using a Student's *t*-test. The significance level was set to $P < 0.05$. Statistical analyses were carried out using Stat-View

software (SAS Institute, NC, USA).
